# Somatic and Abdominal Acupuncture for Pain Treatment in Adolescent Complex Regional Pain Syndrome (CRPS) of the Upper Limb: A Case Report

**DOI:** 10.3390/children8121187

**Published:** 2021-12-16

**Authors:** Giuliano Marchetti, Alessandro Vittori, Ilaria Mascilini, Elisa Francia, Antonella Insalaco, Fabrizio De Benedetti, Sergio Giuseppe Picardo

**Affiliations:** 1 Department of Anesthesia and Critical Care, ARCO Roma, Ospedale Pediatrico Bambino Gesù, IRCCS, Piazza S. Onofrio 4, 00165 Rome, Italy; alexvittori@libero.it (A.V.); ilaria.mascilini@opbg.net (I.M.); elisa.francia@opbg.net (E.F.); sgiuseppe.picardo@opbg.net (S.G.P.); 2 Division of Rheumatology, Ospedale Pediatrico Bambino Gesù, IRCCS, Piazza S. Onofrio 4, 00165 Rome, Italy; antonella.insalaco@opbg.net (A.I.); fabrizio.debenedetti@opbg.net (F.D.B.)

**Keywords:** pain, chronic pain, complex regional pain syndrome, acupuncture, abdominal acupuncture, somatic acupuncture, rheumatology, adolescent, limb, analgesia

## Abstract

Complex regional pain syndrome (CRPS) is still poorly understood. It is a pain disorder in which pain is disproportionate to the initial stimulus. There is no specific therapy for CRPS, but it can be managed by a combination of treatments. We report a 13-year-old girl with CRPS of the upper limb treated with somatic and abdominal acupuncture. She described a severe, pulsating pain in the left wrist and hand, with hypersensitivity, allodynia, a marked reduction in strength, and swelling and sweating at the level of the fingers. Pain began three months previously, after a trauma to the left wrist. The diagnostic tests performed were negative. At the first visit we recommended oral tramadol. During the first two sessions we used somatic acupuncture. At the third session, the girl reported suffering intolerable pain in the affected limb during the previous sessions, so we decided to use abdominal acupuncture. After 8 sessions of abdominal acupuncture the pain completely disappeared. Acupuncture could be a potential alternative when conservative therapy with physical and medical treatment fails, but more often parents and adolescents prefer this therapy since other comorbidities are often present in pediatric populations and abdominal acupuncture could be a valuable alternative aid.

## 1. Introduction

Recently, the International Association for the Study of Pain (IASP) included complex regional pain syndrome (CRPS) among the chronic pain syndromes described in the primary chronic pain group [1,2,3]. CRPS is still poorly understood, and often goes unrecognized or disbelieved and attributed to a particular psychological state of the patient [4]. The term defines a pain disorder in which pain is disproportionate to the initial stimulus. There are three distinct types: complex regional pain syndrome type 1, with pain syndrome, trophic changes and autonomic dysfunction, somatomotor abnormalities without any identifiable peripheral nerve component, and psychological abnormalities (traditional reflex sympathetic dystrophy); complex regional pain syndrome type 2, which includes all the above plus identifiable nerve involvement (classic causalgia); and complex regional pain syndrome type 3 (CRPS with remission of some features) [4,5,6,7]. It is only recently that epidemiological studies on CRPS have been carried out. In 2003, Sandroni et al. in a study conducted in Olmsted County, Minnesota, found the incidence of CRPS1 to be 5.46 per 100,000 people (0.55%), while the incidence of CRPS type II was 0.82 per 100,000 people (0.82%) [8]. There is no specific therapy for CRPS, but symptoms can be managed by a combination of physical treatments, medicaments and psychological support [9]. Moreover, early treatment of CRPS could be beneficial [10].

In this case report, we describe the use of somatic and abdominal acupuncture in a 13-year-old girl with CRPS of the upper limb. Studies conducted with modern methods of Western medicine have postulated that acupuncture analgesia occurs through both the mobilization of central neurogenic peptides, and the stimulation of the central inhibitory pathways of the modulation of painful sensation [11,12].

Abdominal acupuncture is also an ancient technique, but its use has only recently been reintroduced. In accordance with a “turtle representation” of the somatosensory areas in the abdominal region, it is based on the stimulation of the points in this area. Unlike “somatic” acupuncture, in abdominal acupuncture, the needle is superficially driven, there is no need for its stimulation and the treatment is based on the symptom location, thus allowing more standardized protocols [13]. The shallow needles have a diameter of 0.16–0.25 mm, thereby causing less damage and discomfort [14]. Abdominal acupuncture is therefore less painful and more easily applicable in pediatric patients, patients with a weak physical constitution such as the elderly, and patients with immune deficiencies, and is especially indicated for painful or chronic pathologies [15].

## 2. Case Presentation

### 2.1. Evaluation

After a multidisciplinary evaluation, at the end of November 2019, a 13-year-old girl attended the Pain Therapy Clinic of the Ospedale Pediatrico Bambino Gesù in Rome, where acupuncture is also practiced as an analgesic technique. She reported pain in the left wrist and hand, with intensity 10 on the Numeric Pain Rating Scale (NRS), pulsating, and always present, thus preventing any movement. Marked hypersensitivity and allodynia were present at the level of the left fingers, wrist and hand. Pain was present in both flexion and extension of the wrist and caused a marked reduction in strength in the left wrist and hand, making a handshake impossible for the girl. There was no redness but swelling and sweating at the level of the fingers of the left hand. The pain was so intense that it interfered with her regular attendance at school, generating social withdrawal phenomena, which are unfortunately very frequent in patients with chronic pain.

**Figure 1:**
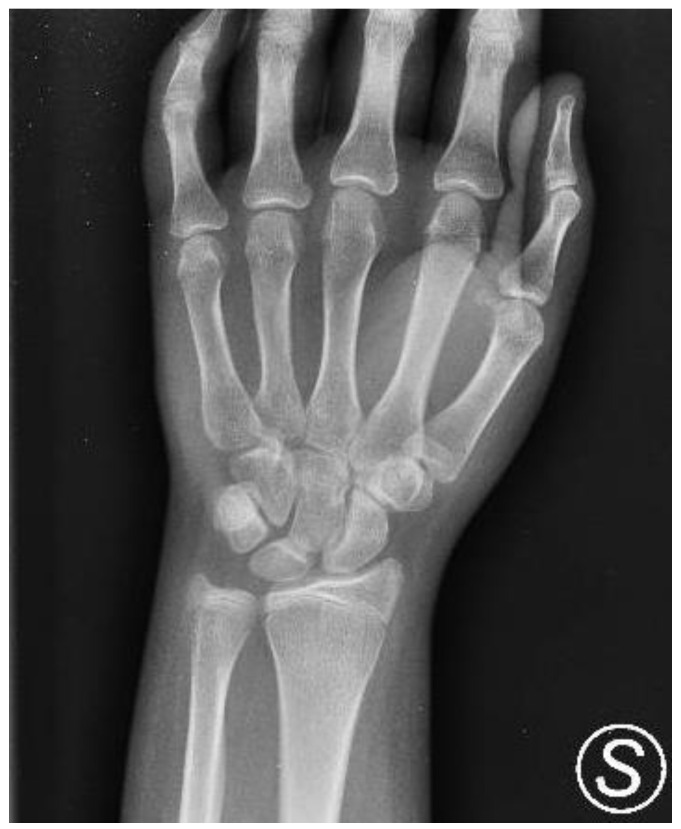
X-ray of the left wrist. Images not attributable to periosteal reaction.

**Figure 2:**
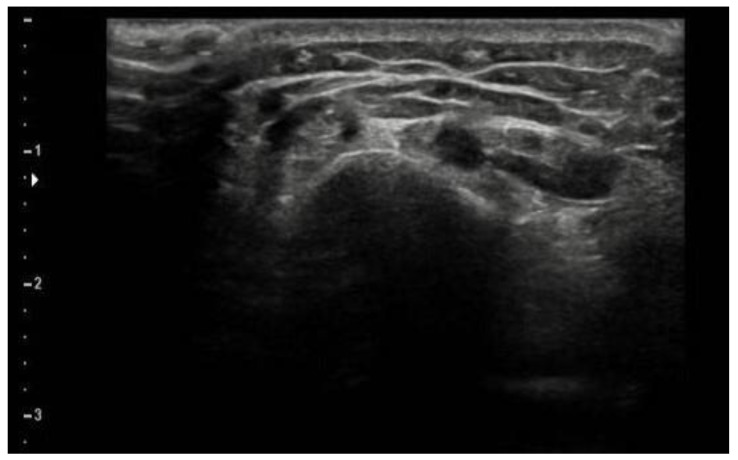
Doppler ultrasound of the wrist: thickening of the subcutaneous soft tissues associated with a minimal flap of effusion in accordance with the tendon sheath of the extensor tendons of the fingers is seen. No further alterations that can be evaluated with this method are observed.

Pain began three months before the consultation, after an accidental fall with trauma to the left wrist. It gradually increased and did not respond to either NSAIDs or limb immobilization. The diagnostic tests performed at the time (X-ray, Doppler ultrasound and magnetic resonance imaging) were negative, as were the blood chemistry tests (Figure 1 and Figure 2).

From the age of 8, the girl was followed by a pediatrician in our hospital for a history of cramps and pain in the lower limbs, especially in the ankle and left knee, both in the absence of trauma or caused by frequent falls. In the following years, clinicians found bilateral flatfoot (with subsequent surgery), mild ligamentous hyperlaxity and vitamin D deficiency. In addition, borderline cognitive level with motor coordination disorder and executive function deficit, as well as stuttering, were then diagnosed. Elements of anxiety emerged from the administration of Self Administered Psychiatric Scales for Children and Adolescents (SAFA-A, D and S) questionnaires, as highlighted in particular by the subscales “Separation anxiety” and “Generalized anxiety” [16,17]. Concern for one’s own health was observed, with experiences of herself as being ill; the scores indicate a propensity to somatize. There was a tone of mood oriented in a deflected sense, and insecurity. The results of the Lie scale were: 8; T: 65 [18,19]. In addition to pulsating headache with phono and photophobia, frontal epilepsy was also diagnosed, which could explain the frequent falls, and moreover had excluded hyperbaric oxygen therapy as a feasible regimen in this case (due to a cost/benefit evaluation, and increased exposure to oxygen toxicity during the treatment itself). Episodes of dizziness with difficulty in maintaining an upright position lasting a few hours were also observed, and on two occasions she also had an episode of unconsciousness lasting about 2 min.

### 2.2. Treatment

During the first visit, as a consequence of the mother’s need to have time to convince the recalcitrant daughter, in the meantime it was recommended to administer oral tramadol (100 mg/mL), 5 gtt in the morning and 5 gtt in order to reduce musculoskeletal pain. The reason for choosing a drug such as tramadol rather than any other pharmacological option lies in the fact that the pain was so intense that it affected the patient’s relationships [20]. We therefore opted for a drug that would have an immediate effect, so as to be able to undertake the acupuncture course. Subsequently, in the first two sessions, after careful disinfection of the skin with 2% chlorhexidine, and using the appropriate needles for length and diameter based on the type of acupuncture and the selected points, we used the following acupoints: TE 4 (Yang Pool), TE 5 (Waiguan), LI 5 (Yang Xi) and SI 4 (Wan Gu), all on the left side. These points were chosen on the basis of a pathology which, according to traditional Chinese medicine, was caused by cold wind. These points produced heat and dissipated the wind [21]. The needles were kept for 30′, with stimulation every 10′. In the third session, the girl reported a slight improvement in the painful symptoms but at the same time unbearable pain in the affected limb during the previous sessions due to the insertion and maintenance of the needles in the affected area. We decided to change strategy and to use abdominal acupuncture and stimulation of the points CV 4 (Guan Yuan), CV 12 (Zhong Wan), CV 16 (Zhong Ting), CV 17 (Shan Zong), ST 24 bilateral (Huaroumen) and KI 17 bilateral (Shang Qu), with appropriate needles and maintaining the same interspeed and time of stimulation. The needles were inserted to a depth of 0.2 cun. These new points (CV 4, CV 12, CV 16, CV 17 and KI 17), in addition to heating and dissipating the wind, re-established the correct circulation of qi [22]. In fact, used in combination, their purpose was to move the qi from the kidney to the extremity of the upper limb passing through the shoulder [21,23]. ST 24 was used to calm the patient and increase her compliance with acupuncture [21].

Auriculotherapy was also associated with Vaccaria seeds on the Wrist, Hand and Shenmen points, with the recommendation to stimulate them at home for 10 min, 4 times a day until the next session. Vaccaria seeds are used to stimulate certain points in auriculotherapy due to their almost spherical shape, and the absence of pharmacological properties [24]. The Shenmen point was chosen for its anxiolytic effect, given the patient’s psychological difficulties [25].

### 2.3. Outcome

After eight sessions (two months) of abdominal acupuncture the pain completely disappeared (Numeric Pain Rating Scale value 0) and the girl regained full functional capacity of the arm and a normal life. Follow-up at three months, six months and one year demonstrated complete remission of symptoms, with constant values of NRS equal to 0. The reduction in pain, and subsequently its total disappearance, allowed the patient to resume normal school attendance, and therefore to resume a life of normal, balanced relationships.

## 3. Discussion

Over the years several diagnostic criteria for complex regional pain syndrome (CRPS) have been proposed: the International Association for the Study of Pain (IASP) criteria and the Budapest diagnostic criteria, introduced in 1994, showed lack of specificity and internal settings [10,26]. The Budapest criteria added stringent criteria to increase the specificity of the diagnosis. At present, CRPS diagnosis is essentially clinical [27].

Our clinical case falls within CRPS type I, following a trauma to the left wrist without any injuries that could justify the clinical picture, as demonstrated by the presence of intense pain, functional limitation, trophic and autonomic and psychological disorders. All investigations carried out in connection with the event or following previous pathological episodes did not reveal any pathologies that could explain this CRPS of the left wrist and hand. Although the diagnosis of CRPS seems to be the most probable, rather than fibromyalgia for example, it is not possible to have a definite diagnosis; this is a limitation of this case [28].

In our case, the psychological approach for the treatment of CRPS was already in place, since the patient was followed by a team of psychologists. However, this treatment, which is a cornerstone of the multidisciplinary approach, was not enough. For this reason, non-pharmacological attack treatment, upon which acupuncture is based, was chosen.

The use of acupuncture in the treatment of CRPS pain is still widely debated. There are very few papers on the subject and these refer to adults only [4].

## 4. Conclusions

There is still no gold standard for the diagnosis of complex regional pain syndrome (CRPS). Clinical history and physical examination form the cornerstones of the diagnostic process [29]. Many conservative approaches have been suggested for the treatment of pain in CRPS and we think acupuncture could have a leading role in this case, as in other cases of chronic pain. However, large multi-center studies on this subject will be necessary to provide evidence of its efficacy [30,31,32,33]. In our patient, somatic acupuncture was replaced by abdominal acupuncture when, during the third session, the girl spoke of her suffering. This allowed us to discover that abdominal acupuncture (possibly in association with somatic acupuncture and auriculotherapy) can be a valuable aid, since in our case we obtained an interesting and stable result over time.

However, further and prospective studies are needed to verify the real efficacy of acupuncture in CRPS.

## Data Availability

Not applicable.
